# Cost-effectiveness of CYP2C19 genotyping to guide antiplatelet therapy for acute minor stroke and high-risk transient ischemic attack

**DOI:** 10.1038/s41598-021-86824-9

**Published:** 2021-04-01

**Authors:** Zeling Cai, De Cai, Ruiwen Wang, Heng Wang, Ze Yu, Fei Gao, Yuansheng Liu, Yingbo Kang, Zhuomin Wu

**Affiliations:** 1 Department of Finance, The First Affiliated Hospital of Shantou University Medical College, Shantou, China; 2 Department of Pharmacy, The First Affiliated Hospital of Shantou University Medical College, No. 57 Changping Road, Shantou, 515041 Guangdong China; 3 The First Affiliated Hospital of Shantou University Medical College, Shantou, China; 4 Beijing Medicinovo Technology Co. Ltd., Beijing, China

**Keywords:** Computer modelling, Neuroscience, Health care

## Abstract

Dual antiplatelet therapy (DAPT) with clopidogrel plus aspirin within 48 h of acute minor strokes and transient ischemic attacks (TIAs) has been indicated to effectively reduce the rate of recurrent strokes. However, the efficacy of clopidogrel has been shown to be affected by cytochrome P450 2C19 (CYP2C19) polymorphisms. Patients carrying loss-of-function alleles (LoFAs) at a low risk of recurrence (ESRS < 3) cannot benefit from clopidogrel plus aspirin at all and may have an increased bleeding risk. In order to optimize antiplatelet therapy for these patients and avoid the waste of medical resources, it is important to identify the subgroups that genuinely benefit from DAPT with clopidogrel plus aspirin through CYP2C19 genotyping. This study sought to assess the cost-effectiveness of CYP2C19 genotyping to guide drug therapy for acute minor strokes or high-risk TIAs in China. A decision tree and Markov model were constructed to evaluate the cost-effectiveness of CYP2C19 genotyping. We used a healthcare payer perspective, and the primary outcomes included quality-adjusted life years (QALYs), costs and the incremental cost-effectiveness ratio (ICER). Sensitivity analyses were performed to evaluate the robustness of the results. CYP2C19 genotyping resulted in a lifetime gain of 0.031 QALYs at an additional cost of CNY 420.13 (US$ 59.85), yielding an ICER of CNY 13,552.74 (US$ 1930.59) per QALY gained. Probabilistic sensitivity analysis showed that genetic testing was more cost-effective in 95.7% of the simulations at the willingness-to-pay threshold of CNY 72,100 (GDP per capita, US$ 10,300) per QALY. Therefore, CYP2C19 genotyping to guide antiplatelet therapy for acute minor strokes and high-risk TIAs is highly cost-effective in China.

## Introduction

Minor strokes and transient ischemic attacks (TIAs) are warning signs of an impending stroke, which is the leading cause of death in China^1^. The early (90-days) risk of recurrence of stroke and serious vascular events following a minor stroke or TIA is particularly high, and it is therefore considered a standard way to measure treatment efficacy^2,3^.

Dual antiplatelet therapy (DAPT) has been shown to be a more effective treatment and prevention strategy in patients with a minor stroke or TIA than aspirin alone^4,5^. Among the options, the combination of clopidogrel plus aspirin is the most well-established antithrombotic strategy. A large number of studies have shown that DAPT with clopidogrel plus aspirin can effectively reduce the risk of stroke recurrence in patients with a minor stroke or TIA; in the international guidelines, the combination of clopidogrel plus aspirin has become the recommended therapy for the secondary prevention of stroke in patients with a minor stroke or acute TIA^6,7^. The combination therapy of clopidogrel plus aspirin is not the only choice for patients with a minor stroke or TIA as dipyridamole plus aspirin offers an alternative strategy. Barlas RS found that the addition of dipyridamole to aspirin had higher short-term efficacy than aspirin alone, which could reduce the mortality rate within 90 days and the recurrence rate of stroke and serious vascular events^8^. According to the American Heart Association/American Stroke Association (AHS/ASA) guidelines for the secondary prevention of a minor stroke or TIA, the sustained-release dosage form of dipyridamole combined with low-dose aspirin is also recommended as an alternative to aspirin and clopidogrel when a contraindication to clopidogrel exists^9^.

Clopidogrel is an adenosine diphosphate (ADP) receptor antagonist and is the only drug in its class to be indicated for the secondary prevention of ischemic cerebrovascular accidents^10^. However, there are significant differences in individuals’ pharmacodynamic response to clopidogrel^11^. Clopidogrel is a prodrug that requires metabolism by cytochrome P450 2C19 (CYP2C19) in the liver into an active metabolite to exert its antiplatelet aggregation effect^12,13^. In addition, CYP2C19 polymorphism leads to individual differences in the pharmacokinetics of clopidogrel. CYP2C19*2, CYP2C19*3 and CYP2C19*17 are three main variant genotypes of CYP2C19. Patients carrying CYP2C19*2 or CYP2C19*3 are classified as moderate metabolizers (CYP2C19*2 or CYP2C19*3) or slow metabolizers (CYP2C19*2 and CYP2C19*3) because their CYP2C19 genotypes are associated with diminished enzyme activity. Therefore, both moderate and slow metabolizers are defined as CYP2C19 loss-of-function allele (LoFA) carriers. Furthermore, CYP2C19*17 results in increased activity, and patients with CYP2C19*17 were classified as fast metabolizers and were defined as CYP2C19 LoFA noncarriers. LoFA carriers have a decreased response to clopidogrel in minor stroke or TIA patients. In fact, apart from CYP2C19 polymorphisms, many other genetic polymorphisms that are relevant to the disposition and efficacy of clopidogrel may influence the pharmacodynamic effects of clopidogrel for patients with an ischemic stroke or a TIA. However, a meta-analysis on the relationship between genetic polymorphisms and clopidogrel efficacy for an ischemic stroke or a TIA in 2016 showed that genetic polymorphisms other than CYP2C19 were not associated with clinical outcomes such as stroke recurrence and composite vascular events among patients with an ischemic stroke or a TIA^14^. Unfortunately, CYP2C19 LoFAs are very common within Asian populations^15,16^. It has been reported that almost 62% of East Asians carry at least one CYP2C19 LoFA (CYP2C19*2 or CYP2C19*3)^17^.

Therefore, clopidogrel resistance and clopidogrel treatment failure rates may be high in East Asians. The Clopidogrel in High-Risk Patients with Acute Nondisabling Cerebrovascular Events (CHANCE) trial showed that the benefit of clopidogrel in patients with an acute minor stroke or a high-risk TIA in China depends on their CYP2C19 genotype and their ESRS score. ESRS is a predictive tool for evaluating the recurrence risk of ischemic stroke patients not caused by atrial fibrillation. The range of ESRs is 0–9 points, where 0–2 points indicates that the recurrence risk of stroke or compound cardiovascular events is lower in the low-risk group, and ≥ 3 points indicates that the recurrence risk of stroke or compound cardiovascular events is higher in the high-risk group (supplemental table). The CHANCE trial showed that LoFA carriers at low risk (ESRS < 3) did not benefit at all from DAPT with clopidogrel plus aspirin, which means that the clinical outcome of clopidogrel plus aspirin is equivalent to the use of aspirin alone. Furthermore, the short-term and long-term safety evaluation showed an increased risk of bleeding among LoFA carriers treated with clopidogrel plus aspirin therapy. For these patients, adjusting the standard DAPT (clopidogrel plus aspirin) to the combination of dipyridamole plus aspirin could optimize the antiplatelet regimen and improve medicine safety^18^, therefore avoiding the waste of medical resources. Therefore, it is important to distinguish patients who are not suitable for DAPT with clopidogrel plus aspirin through CYP2C19 genotyping to guide antiplatelet therapy. However, the cost-effectiveness of CYP2C19 genetic testing, which is relatively costly but important to patients, clinicians and policymakers, has not yet been assessed. The current cost-effectiveness study estimates the economic outcomes of CYP2C19 genetic testing to guide antiplatelet therapy for Chinese minor stroke/acute TIA patients.

## Results

### Base-case analysis

Genetic testing resulted in a gain of 0.031 quality-adjusted life years (QALYs) per patient and an increase of CNY 420.13 (US$ 59.85) compared to the results in the nongenetic testing group. The incremental cost-effectiveness ratio for the treatment of acute minor stroke or high-risk TIA patients in the genetic testing group versus patients in the nongenetic testing group was CNY 13,552.74/QALY (Table 1).

**Table 1:** Cost and QALYs per capita in base-case analysis.

Strategy	Cost (CNY)	QALYs	ICER (CNY/QALY)
Genetic testing regimen	123,869.50	5.1450	13,552.74
Non-genetic testing regimen	123,449.37	5.1140	

*CNY* Chinese Yuan Renminbi, *QALYs* quality-adjusted life years, *ICER* incremental cost-effectiveness ratio.

In the base case, for patients with an acute minor stroke or a high-risk TIA, the genetic testing program would require an additional cost of CNY 420.13 (US$ 59.85) to obtain 0.031 QALY, which is far less than the GDP per capita. Therefore, CYP2C19 genetic testing to guide antiplatelet therapy for patients with a minor stroke or an acute TIA has more cost-effective advantages, and the additional cost is fully merited.

### Sensitivity analysis

**Figure 1:**
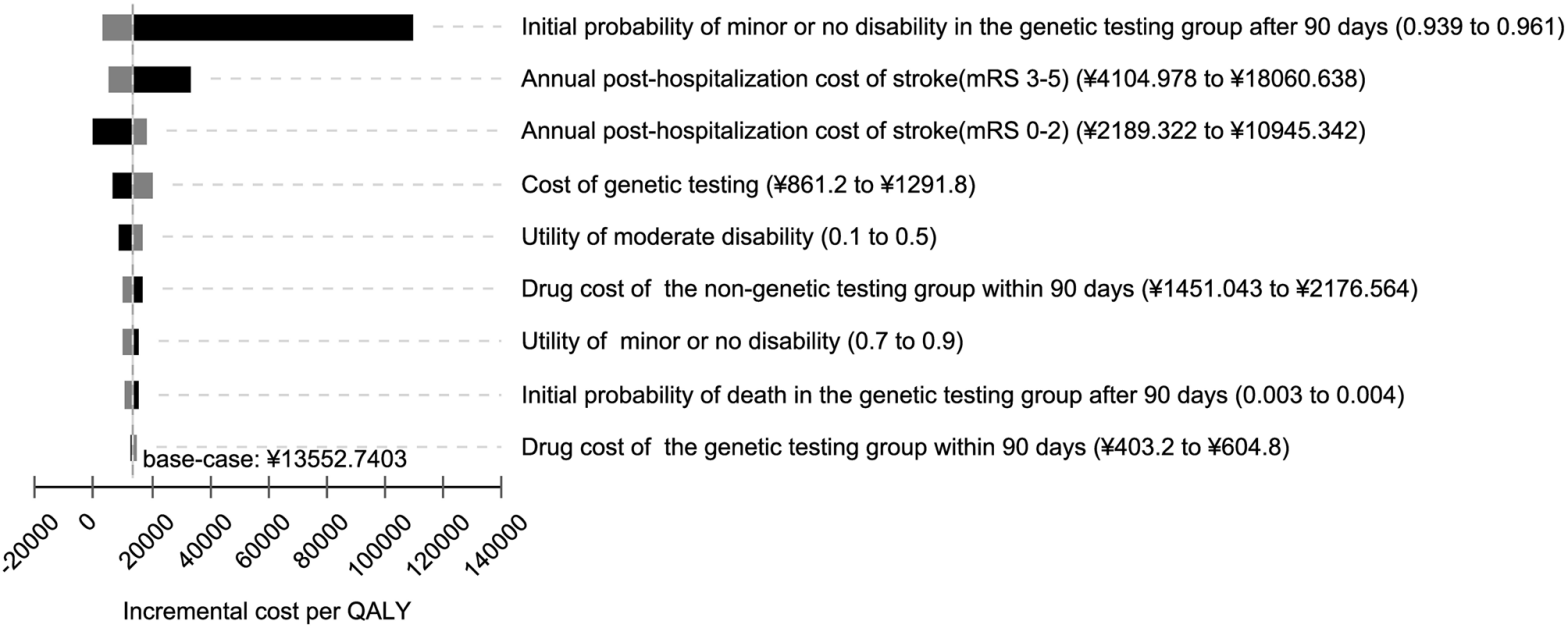
One-way sensitivity analyses on incremental cost-effectiveness ratio (ICER) gained by genetic testing. Numbers listed in parentheses indicate the range of the variable. Dark-shaded bars represent the lower bound of the variable range. Light-shaded bars represent the upper bound. Solid vertical lines represent the ICER of the clopidogrel-aspirin regimen at the base-case scenario (CNY 13,552.74/QALY). QALYs, quality adjusted life years.

The results of the one-way sensitivity analysis are shown in Fig. 1, which showed only the top nine parameters that had the greatest impact on the ICER. Overall, the result is sensitive to the proportion of patients with minor or no disability in the genetic testing group on the 90th d, the annual posthospitalization costs of a stroke (mRS 3–5 and mRS 0–2) and the cost of genetic testing. When the initial probability of minor or no disability in the genetic testing group on the 90th day was decreased to 0.939, the ICER of genetic testing decreased to CNY 109,486.79/QALY, which remains far below the willingness-to-pay threshold (CNY 216,000 per QALY, 2019.). When the annual posthospitalization costs of a stroke (mRS 3–5) varied from CNY 4104.98 to CNY 18,060.64, the ICER of genetic testing increased from CNY 4961.67/QALY to CNY 33,439.13/QALY.

### Probabilistic sensitivity analysis

**Figure 2:**
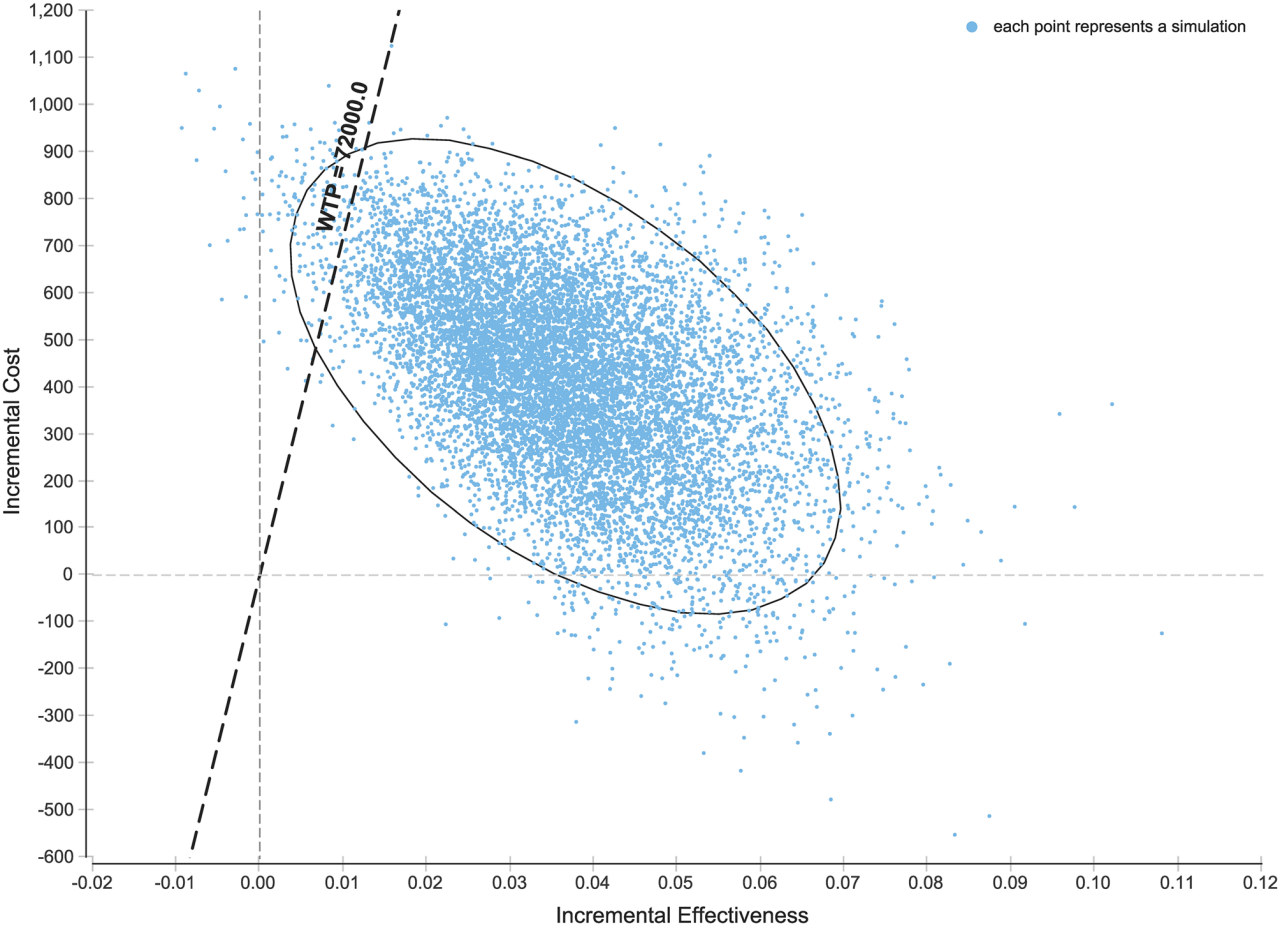
Incremental cost-effectiveness scatterplot of the result of the probabilistic sensitivity analysis. The dashed line represents the willingness-to-pay threshold of CNY 105,000 per QALY. The white square represents the base-case (0.031 QALYs gained at an incremental cost of CNY 420.13). CNY, Chinese Yuan Renminbi; QALYs, quality adjusted life years; WTP, willingness-to-pay.

**Figure 3:**
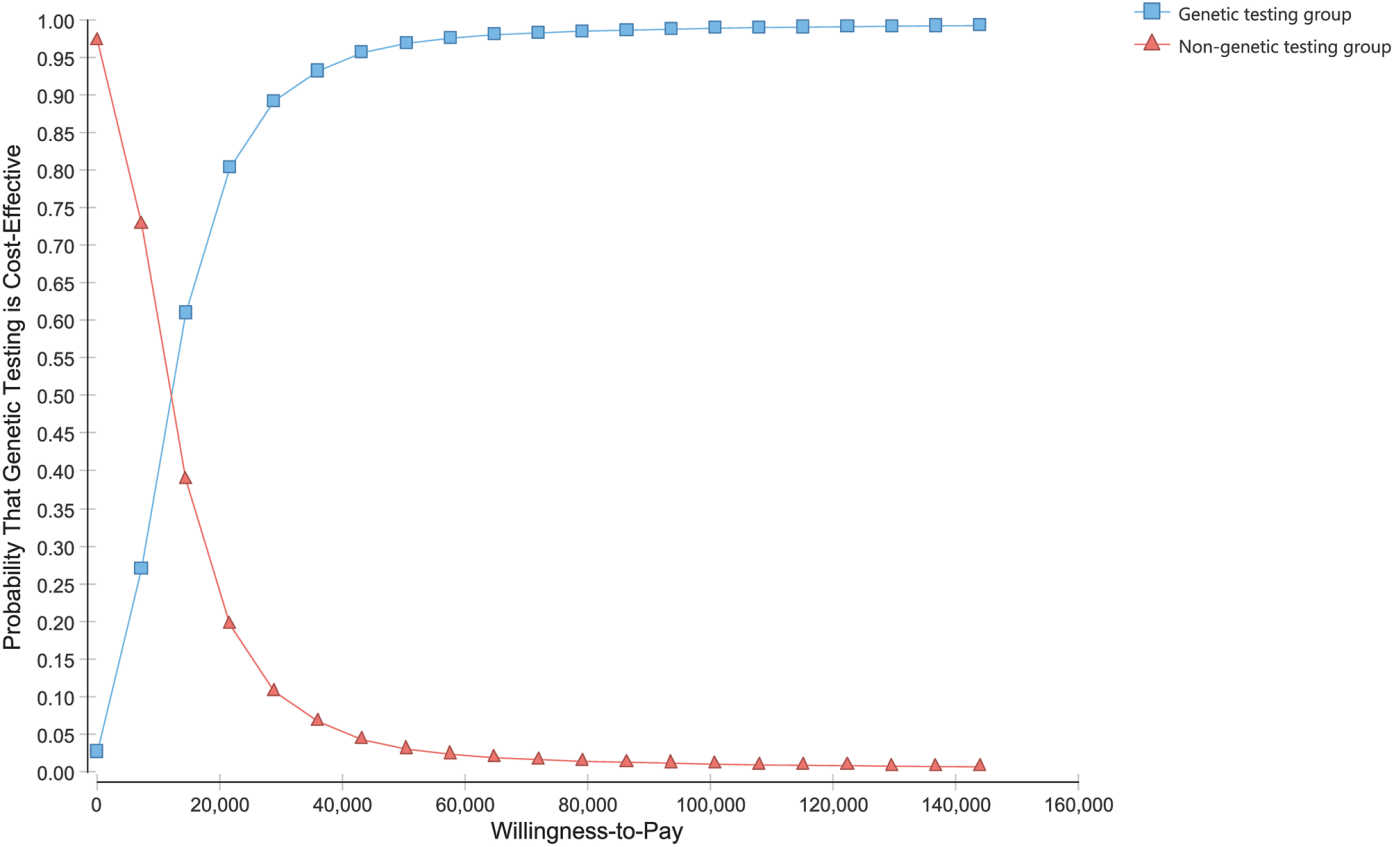
Cost-effectiveness acceptability curve and frontier; y-axis values indicate the probability of a strategy being cost-effective across a wide range of willingness-to-pay thresholds. The solid vertical line represents the willingness-to-pay threshold of CNY 216,000 per QALY. The dashed vertical line represents CNY 72,100 per QALY. CNY, Chinese Yuan Renminbi.

The results of 10,000 iterations of the probabilistic sensitivity analysis are shown in Fig. 2. In 96.94% of the simulations run, genetic testing was beneficial and resulted in more QALYs than nongenetic testing. It remained cost-effective in 95.71% of the simulations at a willingness-to-pay threshold of CNY 72,100 (GDP per capita, US $10,300) per QALY. In 2.59% of the simulations run, genetic testing resulted in benefits in QALYs and cost savings. The cost–benefit acceptability curve shows the relationship between the cost–benefit probability of the genetic testing program and the willingness-to-pay threshold (Fig. 3).

## Discussion

We evaluated the long-term economic outcomes associated with the use of CYP2C19 genetic testing to guide antiplatelet therapy for acute minor stroke or high-risk TIA patients in China using a disease-based decision analytic model. The results of our study suggest that using genetic testing to guide antiplatelet therapy compared with the conventional DAPT strategy (clopidogrel plus aspirin) is a highly cost-effective treatment for acute minor stroke or high-risk TIA patients in China. Specifically, our analyses indicated that for every patient subjected to genetic testing, 0.031 QALY would be gained at a cost of CNY 420.13 (US$ 59.85). The sensitivity analysis showed good stability of the model.

To our knowledge, this is the first analysis of the pharmacoeconomic advantages of CYP2C19 genotyping to guide antiplatelet therapy for acute minor stroke or high-risk TIA patients in China. Wang Yongjun et al. observed the impact of CYP2C19 polymorphism on the antiplatelet effects of clopidogrel plus aspirin in minor stroke/TIA patients. In our study, dipyridamole-aspirin therapy was chosen to replace conventional therapy consisting of clopidogrel plus aspirin for patients who could not benefit from conventional DAPT therapy. The Second European Stroke Prevention Study (ESPS 2) found that the addition of dipyridamole (extended release 200 mg twice daily) to aspirin (50 mg daily) reduced serious vascular events by 22% in comparison with aspirin alone^19,20^. Halkes PH et al. further analyzed 7612 patients in 5 trials and found that dipyridamole-aspirin was more effective than aspirin in the secondary prevention of stroke in minor stroke or TIA patients^21^. Most importantly, however, according to the guidelines for the secondary prevention of cerebral infarction or TIA updated by AHS/ASA in 2014, sustained-release dipyridamole in combination with low-dose aspirin is recommended as a substitution therapy for clopidogrel-aspirin when a contraindication to clopidogrel exists^22,23^. In this study, we analyzed the economic efficiency of only one alternative therapy for patients who would not benefit from conventional DAPT. Ticagrelor plus aspirin was not considered as a substitute strategy because of a lack of sufficient supporting evidence^24^. Moreover, ticagrelor has not been approved for the treatment and secondary prevention of acute ischemic strokes and TIAs in China. Furthermore, patients with an acute minor stroke or a TIA who were CYP2C19 LoFA carriers were not advised to increase their dose of clopidogrel because there is insufficient evidence to support this practice. The treatment strategies in our study strongly illustrated the significant benefits of genetic testing in these patients.

One-way sensitivity analysis indicated that the study results were reliable. Cost-effectiveness was sensitive to the proportion of patients with minor or no disability in the genetic testing group on the 91st day, the annual posthospitalization costs of a stroke (mRS 3–5 and mRS 0–2) and the cost of genetic testing. This indicated that the sensitivity and specificity of gene detection may greatly influence the model results. Based on the published literature, we assumed that the initial probability range of disability status in the genetic testing group was 0.939–0.961, within which the ICER of the genetic testing remained far below the willingness-to-pay threshold. When the annual posthospitalization costs of patients with moderate or severe disability (mRS 3–5) was reduced to approximately a quarter of the base case level, the cost savings from genetic testing decreased, and the ICER was CNY 4961.67, which was still substantially below the willingness-to-pay threshold. To ensure that the data are closer to the actual situation in China, after the cost of genetic testing was adjusted from CNY 861.2 to CNY 1291.8, genetic testing was still beneficial. Our study shows that using genetic testing to guide the treatment of minor stroke or TIA patients is more cost-effective than direct DAPT.

There are several limitations of our study worth noting. Female patients accounted for only 33% of the total CHANCE trial population to which we referred, which is not consistent with the clinical rates of a minor stroke or a TIA and may result in different incident rates and medical costs. In consideration of the racial differences in the distribution of CYP2C19 genetic polymorphisms, we only included parameters extracted from a Chinese population, although this decision also makes it difficult to extrapolate our results to other populations. Finally, morbidity varies greatly during the course of treatment depending on the patient’s condition and their treatment. In summary, CYP2C19 genotyping to guide antiplatelet therapy for a minor stroke or acute TIA in China is highly cost-effective, and the findings provide a basis for clinical treatment decisions.

## Methods

### Model overview and structure

The CHANCE study is a double-blind, placebo-controlled, multicenter study conducted in 114 centers from 2008 to 2011 and included 5170 patients with minor stroke or acute TIA in China. The present study relied on the CHANCE trial, which classified patients according to their CYP2C19 polymorphism and ESRS, to determine the appropriate antiplatelet treatment for an acute minor stroke or a TIA. The genetic testing was higher than 98.5%^18^. CYP2C19 genotypes were mainly divided into a fast metabolizer group (CYP2C19*17), a moderate metabolizer group (CYP2C19*2 or *3) and a slow metabolizer group (CYP2C19*2 and *3). The fast metabolizer group was defined as CYP2C19 LoFA noncarriers, and the moderate metabolism group and slow metabolism group were defined as CYP2C19 LoFA carriers. The ESRS scale is a predictive tool for evaluating the risk of recurrence in patients with an ischemic stroke not caused by atrial fibrillation; it ranges from 0 to 9, with 0–2 signifying low risk and ≥ 3 signifying high risk^25^. The proportion of patients with CYP2C19 LoFA and the percentage of patients at high risk for a recurrent stroke (ESRS ≥ 3) were extracted from the genetic substudy of the CHANCE trial (Table 2). After undergoing CYP2C19 genetic testing, CYP2C19 LoFA noncarriers, CYP2C19 LoFA carriers but with an ESRS ≥ 3, and those patients without genetic testing were given clopidogrel-aspirin treatment (clopidogrel: a loading dose of 300 mg followed by 75 mg daily for 3 months; aspirin: a loading dose of 75–300 mg followed by 75 mg daily for 21 days). The antiplatelet strategy for CYP2C19 LoFA carriers and ESRS < 3 was changed to a dipyridamole-aspirin sustained-release capsule (25 mg/200 mg, twice a day) for 90 days. All patients were treated with aspirin alone after 90 days because dual antiplatelet therapy with clopidogrel plus aspirin is not recommended in the long-term treatment of a minor stroke or TIA, and aspirin alone is more common than clopidogrel alone in secondary stroke prevention^26,27^. No human participants were involved in the current study.

**Table 2:** Efficacy of Genetic testing regimen for minor stroke and acute TIA patients.

Model input		Genetic testing		Non-genetic testing	References
Proportion of patients after 90 days					^19,32^
Minor or no disability (mRS 0–2)		0.9506		0.9376	
Moderate disability (mRS 3–4)		0.0404		0.0527	
Severe disability (mRS 5)		0.0054		0.0058	
Death (mRS 6)		0.0036		0.0039	
**90-day event risks**	CYP2C19 LoFA non-carriers*	CYP2C19 LoFA carriers^†^ and ESRS ≥ 3	CYP2C19 LoFA carriers and ESRS < 3		^19,21,32^
Recurrent stroke	0.0717	0.0871	0.0727	0.082	
Proportion of ICH	0.0377	0.0377	0.0377	0.0377	
Major ECH	0.0108	0.0108	0.0108	0.0108	
MI	0.0012	0.0012	0.0012	0.0012	

*Minor stroke* minor stroke, *TIA* transient ischemic attack, *mRS* modified Rankin Score, *ICH* intracerebral hemorrhage, *ECH* extracranial hemorrhage, *MI* myocardial infarction.

*CYP2C19 LoFA non-carriers were defined as patients without LoFA of *2 and *3 (*1/*1, *1/*17, or *17/*17).

^†^CYP2C19 LoFA carriers were defined as patients with at least one LoFA of *2 or *3 (*1/*2, *1/*3, *2/*2, *2/*3, *3/*3, *2/*17, or *3/*17).

**Figure 4:**
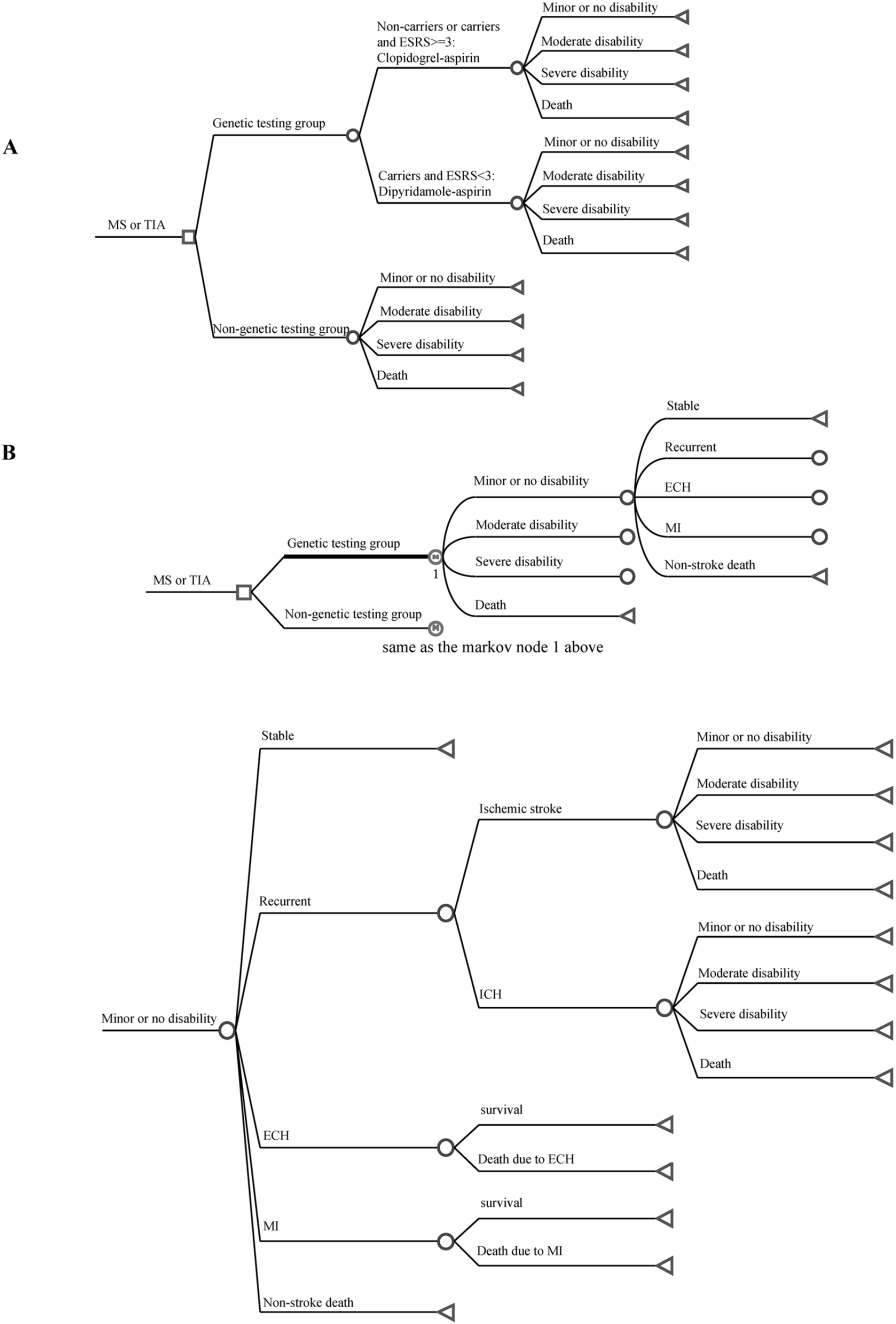
Model structure of cost-effectiveness analysis. (**A**) is the decision tree model within 90 days, and (**B**) is the long-term Markov model. Locations in the model where prescribers can make a decision (squares), chance nodes that are under the control of transition probabilities (circles), and terminal nodes (triangles) are presented. Transitions to future health states leading from the other health states are the same as for the minor or no disability branch. *ECH* extracranial hemorrhage, *ICH* intracerebral hemorrhage, *MI* myocardial infarction.

A decision tree and a Markov model were constructed to evaluate the 90-d and long-term (post-30-year) incremental cost-effectiveness of using CYP2C19 genetic testing to guide antiplatelet therapy for acute minor stroke and high-risk TIA patients (Fig. 4). The Markov model shows the potential transitions between health states for the remaining 30 year^28,29^. In our model, there were four health states based on the modified Rankin Scale (mRS): minor or no disability (mRS 0–2), moderate disability (mRS 3–4), severe disability (mRS 5) and death (mRS 6). All patients entered the decision tree with a health state of minor or no disability. Due to recurrent stroke or severe complications (major extracranial hemorrhage or myocardial infarction), the 90-d short-term outcomes could be minor or no disability, moderate disability, severe disability or death. The data for patients surviving to 90 days in the decision tree were then entered into a Markov model to simulate 30 yr of clinical outcomes.

The analysis was conducted from a healthcare payer perspective using QALYs as an effect indicator. All costs were converted to 2019 Chinese Yuan Renminbi (CNY) by using the medical consumer price index^30^, and long-term costs and outcomes were discounted at 3% per year^32^. The costs of the 90-d consumption of clopidogrel, aspirin and the dipyridamole-aspirin sustained-release capsule were based on the highest retail price in Guangdong. The model was programmed using TreeAge Pro 2019 (TreeAge Software, Inc.).

### Probabilities

In this study, the model input parameters were derived from the results of the CHANCE trial and the published literature, as shown in Table 3. According to the study of Wang et al., patients with CYP2C19 LoFA and at high risk (ESRS ≥ 3) can still significantly benefit from DAPT with clopidogrel plus aspirin at one year. According to the studies of Wang et al., the incidence rates of recurrent stroke were 8.3% and 10.3% in the clopidogrel-aspirin group at 90 days and at one year, respectively^18,31^. In other words, among the patients who received clopidogrel-aspirin therapy and who suffered a recurrent stroke within one year, 80.6% of them occurred within 90 days. Similarly, 81.4% of recurrent strokes occurred within 90 days among those patients receiving aspirin monotherapy. We then assumed that the proportion of recurrent strokes that occurred within 90 days was the same among patients with different metabolic phenotypes in each therapy group. Therefore, the recurrent stroke rates at 90 days were approximately 8.705% and 9.320% in CYP2C19 LoFA carriers but ESRS ≥ 3 in the clopidogrel plus aspirin group and in CYP2C19 LoFA carriers but ESRS < 3 in the aspirin monotherapy group, respectively. Additionally, the recurrent stroke rate at 90 days in CYP2C19 LoFA noncarriers in the clopidogrel plus aspirin group was obtained directly from the results of the CHANCE trial. A meta-analysis showed that the rate of recurrent stroke in patients treated with aspirin and dipyridamole as antiplatelet therapy was lower than that in patients treated with aspirin monotherapy at HR 0.78 (95% CI 0.68–0.90)^21^. Accordingly, we were able to calculate the recurrent stroke rate in CYP2C19 LoFA noncarriers but ESRS < 3 who received aspirin plus dipyridamole therapy. It was then reported that DAPT might reduce stroke-related disability in minor stroke or TIA patients compared with aspirin monotherapy (OR 0.78, 95% CI 0.62–0.99)^21^. Considering that the clinical outcomes of patients experiencing mild or moderate disability due to stroke are always significantly better than those who experience a severely disabling stroke, we further hypothesized that DAPT mainly reduced the proportion of patients experiencing moderate disability (mRS 3–4) and increased the proportion of patients experiencing minor or no disability (mRS 0–2). We defined the proportion of moderate disability due to stroke recurrence as the “disability rate”. From the above, after adjustment for the treatment of CYP2C19 LoFA carriers but ESRS < 3 (approximately 37% of all the patients), the disability rate of the genetic testing group was further reduced compared with that of the nongenetic testing group. After we distinguished the population that could obtain a tangible benefit from DAPT, we calculated the disability rate due to stroke recurrence in the genetic testing group using the formula below. The distribution of disability status of patients treated with aspirin alone was directly based on the results from the CHANCE trial while in the genetic testing group, the distribution of disability status at 90 days was mainly dependent on the rates of stroke recurrence and the disability rates among different subgroups (Table 2). The short-term risks of myocardial infarction (MI) and major extracranial hemorrhages (ECH) were derived from the results of the CHANCE trial. To simplify the model, we assumed that patients in different subgroups in the genetic testing group had the same short-term risks of MI and major ECH. Furthermore, MI or ECH could not change a patient’s disability status in our model. In the Markov model, we assumed that the stroke recurrence rates were equal across all disability categories^28^ and that there was a 1.017-fold increase in stroke recurrence rates per year^32^. Patients who survived recurrent stroke events were reallocated equally among the disability statuses of equal and greater disability levels. Age-specific mortality rates for nonstroke death were based on the study by Pan et al.

**Table 3:** Model Parameters and the Range of Values Tested in Sensitivity Analyses.

Model input	Base-case	Range reference	References
**Cost inputs (2019 CNY)**			^32^
**One-time hospitalization costs**			
Ischemic stroke, (mRS 0–2)	12,499.91	7,021.54–15,861.18	
Ischemic stroke, (mRS 3–6)	18,700.45	9,711.31–24,627.33	
ICH, (mRS 0–2)	14,086.15	7,515.65–17,613.39	
ICH, (mRS 0–2)	18,700.45	9,711.31–24,627.33	
ECH	9125.97	5,563.25–18,330.50	
MI	21,276.20	8,292.31–37,291.96	
**Annual posthospitalization costs**			
Stroke, (mRS 0–2)	8767.42	2189.32–10,945.34	
Stroke, (mRS 3–5)	13,850.50	4104.98–18,060.64	
**90-day**			
Clopidogrel-aspirin price	490.45	± 20%	
Dipyridamole-aspirin price	504	± 20%	
**Cost of genetic testing**	1,076.5	± 20%	
**Utility inputs**			
Minor or no disability (mRS 0–2)	0.75	0.70–0.90	
Moderate disability (mRS 3–4)	0.39	0.10–0.5	
Severe disability (mRS 5)	0.2	0.00–0.32	
Death (mRS 6)	0	0.00–0.00	
Utility of major ECH	0.8	0.79–0.84	
Utility of MI	0.84	0.67–0.96	
**Probabilities inputs**			
Recurrent rate of stroke (per patient year)	0.1219	0.1163–0.1276	
Among patients with recurrent stroke, proportion with:			
ICH	0.075	0.075–0.146	
Death	0.1933	0.1737–0.2128	
Relative risk of stroke recurrence per life-year	1.017	1.013–1.022	
Age-specific nonstroke death rate	0.0089–0.1654		
Rate of major ECH	0.0038	0.003–0.0048	
Mortality rate of major ECH	0.06	± 20%	
Rate of MI	0.0085	0.006–0.0114	
Mortality rate of fatal MI	0.15	0.103–0.246	
**Discount rate inputs**			^32^
Costs	0.03	0.03–0.08	
Outcomes		± 20%	

*CNY* Chinese Yuan Renminbi, *ICH* intracerebral hemorrhage, *ECH* extracranial hemorrhage, *MI* myocardial infarction, *mRS* modified Rankin Scale.

Formula:

$${\text{Disability}}\,{\text{rate}}\,{\text{due}}\,{\text{to}}\,{\text{recurrence}}\,{\text{in}}\,{\text{the}}\,{\text{genetic}}\,{\text{testing}}\,{\text{ group}} = {\text{A}}*\left( {{\text{B}} - {\text{C}}} \right)/{\text{B}}*\left( {{1} - {\text{C}}} \right)$$

A: the rate of moderate disability due to recurrence in the nongenetic testing group, B: the relative risk ratio of disability rates treated with DAPT versus aspirin monotherapy, C: the proportion of patients who would not benefit from aspirin plus clopidogrel.

### Health utility

The health utilities estimated for the different mRS categories of stroke survivors were derived from the literature^32^. Two major complications, MI and major ECH, were considered temporary health states; patients entering either of these two health states had a short-term disutility of 2 week for major ECH and 30 days for MI unless they resulted in death.

### Sensitivity analysis

To estimate the uncertainty of each input parameter in the model results, deterministic one-way sensitivity analysis was used. The reasonable range of all input parameters, including state probabilities, utility values, and costs, was obtained from the literature or estimated by a variation of 20% above and below each direction. We also used a Monte Carlo simulation to perform probabilistic sensitivity analysis. In this study, the cost-effectiveness and probability followed a normal distribution, triangular distribution, and exponential distribution, respectively. All model parameters were combined over 10,000 simulations to calculate the 95% credible interval for each incremental result using a previously specified distribution that approximates its range. The probabilistic results were plotted on the cost-effectiveness plane, and the cost-effectiveness acceptability curve was generated to show the probability that genetic testing was more cost-effective than nongenetic testing under different willingness-to-pay thresholds.

According to the recommendation of the World Health Organization (WHO) on drug evaluation, if ICER < gross domestic product (GDP = $10,276) per capita, then the increased cost is completely worthwhile; if ICER is between 1 and 3 times the GDP per capita, then the increased cost is acceptable; and if ICER > 3 times the GDP per capita, then the increased cost is not worthwhile. Based on the data released by the National Bureau of Statistics (NBS) on January 17, 2020, the per capita GDP in 2019 was approximately CNY 72,100.

## Supplementary Information

Supplementary Table.

## Data Availability

The authors report that data could be available upon request.
