# Supplementary material for: Cost-effectiveness of CYP2C19 genotyping to guide antiplatelet therapy for acute minor stroke and high-risk transient ischemic attack
Source: Sci Rep. 2021 Apr 1;11:7383. doi: 10.1038/s41598-021-86824-9 (PMC8016871; doi:10.1038/s41598-021-86824-9)
Supplement: Supplementary file 1 — Supplementary Table. [file 41598_2021_86824_MOESM1_ESM.docx]

**Title**

Cost-Effectiveness of CYP2C19 Genotyping to Guide Antiplatelet Therapy for Minor Stroke and Transient Ischemic Attack

**Authors**

Zeling Cai*^1^*, BS; De Cai, BS*^2^*; Ruiwen Wang*^4^*, MS; Heng Wang*^4^*, PhD; Ze Yu*^4^*, MS; Fei Gao*^4^*, MD; Yuansheng Liu, MD*^3^*; Yingbo Kang, MS*^2^*; Zhuomin Wu, MD(🖂)*^2^*

*^1^ Department of Finance, The first Affiliated Hospital of Shantou University Medical College, Shantou, China; ^2^* *Department of Pharmacy, The first Affiliated Hospital of Shantou University Medical College, Shantou, China; ^3^ The first Affiliated Hospital of Shantou University Medical College, Shantou, China; ^4^ Beijing Medicinovo Technology Co. Ltd., Beijing, China*

**Corresponding author**

Zhuomin Wu

Address: No.57 Changping Road, Shantou, Guangdong, 515041, China

Phone: +0754-88905343

Email: [wuzhuomin_2012@163.com](mailto:wuzhuomin_2012@163.com)

**Supplementary material 1**

The Essen Stroke Risk Score (ESRS) was developed to predict 1-year risk of recurrent stroke. The parameters of ESRS and points assigned to these parameters are detailed below:

| The Essen Stroke Risk Score (ESRS) | | |
| --- | --- | --- |
| Risk Factors | Points | Risk groups |
| Age: |  | Low-risk group:(0-2 points); High-risk group:(≥3 points) |
| <65 years | 0 |  |
| 65-75 years | 1 |  |
| >75 years | 2 |  |
| Hypertension | 1 |  |
| Diabetes mellitus | 1 |  |
| Previous myocardial infarction | 1 |  |
| Other cardiovascular disease (except myocardial infarction and atrial fibrillation | 1 |  |
| Peripheral artery disease | 1 |  |
| Smoking | 1 |  |
| Additional TIA or ischemic stroke in addition to a qualifying event | 1 |  |
